# Digital healthcare services in community pharmacies in Switzerland: Pharmacist and patient acceptability, and pharmacist readiness–the Pneumoscope™ pilot study

**DOI:** 10.1177/20552076241313164

**Published:** 2025-01-15

**Authors:** Claudine Backes, Coralie Godot, Cédric Yan Gujer, Noémie Obegi, Alexandre Perez, Alain Gervaix, Marie P. Schneider

**Affiliations:** 1 School of Pharmaceutical Sciences, 27212 University of Geneva, Geneva, Switzerland; 2 Institute of Pharmaceutical Sciences of Western Switzerland, 27212 University of Geneva, Geneva, Switzerland; 3 Division of Paediatric Emergency Medicine, Department of Women, Child and Adolescent, Geneva University Hospitals (HUG), 27212 University of Geneva, Geneva, Switzerland

**Keywords:** Triage, Pneumoscope™, respiratory symptoms, COVID-19, artificial intelligence, community pharmacist, interprofessionality, primary care, digital healthcare

## Abstract

**Background:**

The integration of artificial intelligence (AI)-based pharmaceutical services in community pharmacy (CP) settings has the potential to enhance point-of-care services and improve informed patient access to healthcare. The Pneumoscope™, an innovative AI-powered digital stethoscope that analyses lung sounds to detect specific respiratory pathologies, could be a valuable tool for pharmacists in conducting respiratory screening. To understand how this device can be implemented in the healthcare system, this exploratory research aims to assess the acceptability of pharmacists and patients, and the pharmacists’ readiness to use the Pneumoscope™ in CPs for respiratory disease management.

**Methods:**

A 2-stage exploratory approach was conducted using mixed methods: 1) a qualitative analysis of pharmacists’ acceptability and readiness was developed using semi-structured interviews and focus groups ; 2) followed by a quantitative cross-sectional survey of patients’ acceptability of the device in CPs.

**Results:**

Pharmacists were generally positive about the integration of e-health services into their daily clinical practice, recognizing their potential to improve advanced pharmaceutical triage and collaboration with physicians. Most patients were satisfied with the care provided by CPs, and their acceptability to use the Pneumoscope™ was significantly associated with their level of confidence in AI (p = 0.0092) and with the location of their CP (p = 0.0276).

**Conclusions:**

Digital devices such as the Pneumoscope™ have the potential to reinforce the pharmacists’ clinical roles within an interprofessional team and improve patient care, but further scientific evaluation and implementation are necessary to support its integration and ensure its reimbursement by health insurers.

## Introduction

Respiratory diseases are among the most frequent pathologies in the world, where asthma and Chronic Obstructive Pulmonary Disease (COPD) are the leading ones in Switzerland.^1–3^ Risk factors associated to respiratory disease are constantly rising, especially when considering associations to air pollution.^4^ At latest with the onset of COVID-19 pandemic, respiratory pathologies have been in the spotlight. Worldwide, 773 million cases were reported in December 2023 with 7 million associated death cases.^5^ The COVID-19 outbreak has caused an unprecedented disruption to European healthcare systems, sparing no one. Huge pressure was put on healthcare effectiveness, demanding urgent needs for innovative implementation of early-detection and triage methods in primary healthcare settings. COVID-19 impacts will last for long after the pandemic fades away, creating new patterns of unmet healthcare needs including the effective implementation of digital health services, the development of new strategies ensuring safe hospital journeys and the reduction of shortage in human resources by setting up novel triage systems.^6–8^

Effective triage and early detection implemented in decentralised health services could reduce pressure on hospital emergencies, healthcare systems and save lives.^9^ In complex environments such as primary health care, challenges include priorities identifications and setting up of strategies guiding towards an adapted healthcare system transformation. In Switzerland, community pharmacies (CPs) are an integral part of the healthcare system offering various primary healthcare services, as prevention screening, medication reconciliation, tailored therapeutic education and support in medication adherence and understanding.^10–14^ In addition, pharmaceutical care services and measurements of clinical parameters such as blood pressure, glycemia or lung capacity, can be carried out in community pharmacies to complement unavailable medical care.^15^ Services provided by community pharmacists are well accepted when they are known to the population, with the geographical location of healthcare services playing a significant role. Rural community pharmacies are most often seen as a first line of healthcare access point and consequently, pharmaceutical services are more often and longer used in these areas compared to those offered by urban CPs.^16^ Especially for long-term diseases as chronic respiratory diseases, CPs may have a huge potential to support disease triage and follow up, during pandemic crises and beyond.^8,11^

The screening and follow-up of chronic respiratory diseases such as asthma, especially in children, vary largely depending on their nature and presence of pathological sounds identified. The standard binaural stethoscope, used by medical practitioners to detect abnormal chest sounds, relies on subjective assessment and experience, and is linked to high diagnostic variabilities.^17,18^ Respiratory diseases represent an important medical and cost burden for public health. Using a theory-based simulation model, it has been estimated that COPD will cost the world economy US$ 4326 trillion between 2020 and 2050.^19^ To decrease the medical cost and disease burden, more efforts should be made to treat uncomplicated respiratory diseases on outpatients as potentially leading to significant resource savings.

**Figure 1.**
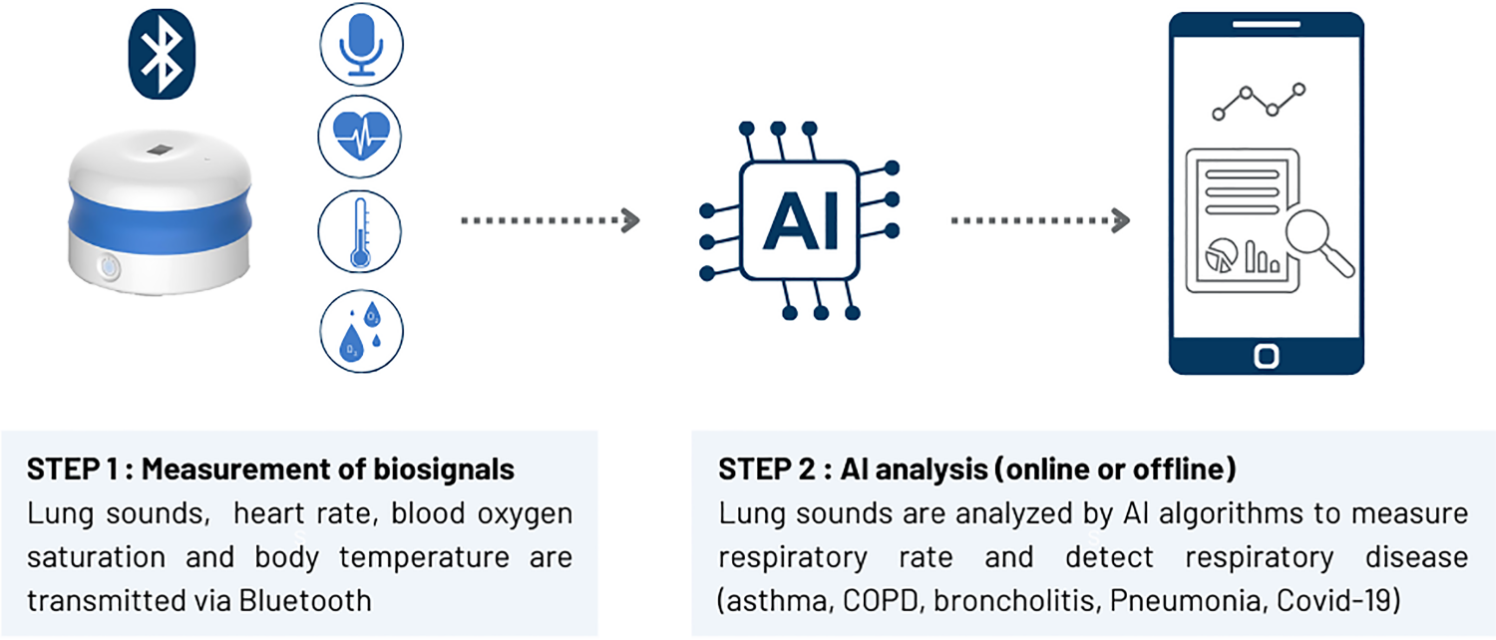
Pneumoscope™ workflow from recording lung sounds, analysing them with AI and transferring the results to a smartphone or tablet.

The era of new healthcare devices using artificial intelligence (AI) is expanding all over the world. The idea of the Pneumoscope^TM^, an intelligent stethoscope, was born in the Paediatric Emergency Department of the Geneva University Hospitals aiming to analyse recorded respiratory sounds by AI to identify normal or abnormal sounds and if abnormal, to classify “audio signatures” in specific respiratory diseases, such as asthma, pneumonia, bronchiolitis or COVID-19 infections.^20^ According to the European regulation, this medical device is classified as a Class IIA hardware device, which presents a low risk to the human body.^21^ This device records and analyse sounds emitted by the respiratory tract at 8 different chest locations (Figure 1).

Thanks to AI algorithms, risk probabilities are provided by the Pneumoscope™ for various respiratory pathologies via a smartphone or an electronic tablet.^22,23^ In a study performed in 572 paediatric outpatients across 5 countries, the AI model (i.e., DeepBreath) used by the Pneumoscope™ differentiated healthy and pathological breathing with an Area Under the Receiver-Operator Characteristic (AUROC) of 0.93 (standard deviation [SD] ± 0.01 on internal validation). Similarly, the accuracy for the classification of diseases was AUROC 0.75 ± 0.10 for pneumonia, AUROC 0.91 ± 0.03 for wheezing disorders, and AUROC 0.94 ± 0.02 for bronchiolitis.^24^ Compared to subjective binaural auscultations, the Pneumoscope™ provides objective findings and thus, optimizes the quality and speed of screening.^23^ The Pneumosope™ may be of great support for patients by avoiding emergency room visits, for use of on-call duty services or to decentralized healthcare services. However, such promising digital healthcare devices only have an impact on health outcomes in case their implementation into the healthcare system is successful and accepted by patients and healthcare workforces. Among the identified factors influencing the implementation of digital health devices, common factors include costs, usability, compatibility with other technologies and versatility of the product, data protection and adaptability to the healthcare contexts.^25,26^ Therefore, it is essential to study the Pneumoscope™ implementation into the healthcare system, to prepare implementation strategies and to develop adapted healthcare services and test them.^25^ The Pneumoscope™ can help to ensure patient timely access to healthcare, increase the accuracy of respiratory disease screening and triage, and reduce healthcare costs.

This study aims to explore acceptability and readiness to use the Pneumoscope™. The acceptability is defined as the user satisfaction and how positively the service is perceived,^27^ while the readiness examines whether the intervention can be effectively implemented considering factors like engagement, availability of resources and knowledge.^28,29^ Thus, this study investigates the first stages of the potential implementation of the Pneumoscope™ in CPs by (i) analysing community pharmacists’ acceptability and readiness on using the Pneumoscope™ for respiratory disease management, by (ii) describing the typology of patients who enter a CP in the French speaking part of Switzerland and by (iii) evaluating patients’ acceptability of digital health services including AI devices as the Pneumoscope™ when offered by CPs.

## Method

### Study design

To ensure a global understanding of the research problem, this exploratory cross-sectional study was conducted using mixed methods, incorporating sequential qualitative and quantitative data collection and analyses.^30–32^

The qualitative analysis was conducted by NO, a Master student in pharmaceutical sciences supervised by MS and CB between July and November 2020 in the French-speaking part of Switzerland. The participants of the study knew that NO was a Master student at the University of Geneva and that she was conducting a study on an AI device in CPs. In the first step, individual semi-structured interviews were conducted with community pharmacists to capture contextual factors, using either telephone or videoconference based upon pharmacists’ preferences. In the second step, the Pneumoscope™ prototype was presented during virtual focus group meetings performed by video conference, to which pharmacists having participated in the first step were invited.

The quantitative analysis, as third step, occurred in CPs in the Canton of Geneva, Switzerland and was conducted by CGU between August and September 2021.

### Study population and sampling

**Qualitative analysis**: Pharmacists working in an independent CP or a pharmacy chain were contacted by e-mail, followed by a telephone call, to participate in the individual interviews. Both pharmacy managers and assistant pharmacists were included to obtain a representative diversity of opinions, including a balance of men, women, age groups and pharmacy geolocation (rural/city) from the French-speaking part of Switzerland. There were no exclusion criteria. For the focus groups, pharmacists were recruited by email among the pharmacists having participated in the individual interview. In case of no reply, a reminder was sent after two weeks.

**Quantitative analysis:** CPs included in the previous qualitative analysis, and new ones, all located in Geneva, were invited via email to volunteer for the third step of the study. People visiting these CPs were invited by a Master student (CGU) to fill in the survey. Patients’ eligibility criteria were French-speaking adults or parents of children leaving their CP after being cared for by the pharmacist and their team. There were no other exclusion criteria. Volunteers provided written explicit consent before participating in the study and the data collection was anonymous.

As the study was exploratory, no sample size was defined. Our aim was to include around one hundred participants to provide the best possible representation of patients visiting CPs.

### Data collection

**Qualitative analysis:** An interview guide (Appendix 1a) was developed for the individual interviews. It was reviewed by all co-authors. It aimed at understanding pharmacists’ cognitive reasoning and management of patients with acute or chronic asthma, respiratory infections, and COVID-19 in community pharmacies. Additionally, community pharmacists’ acceptability and readiness regarding the use of novel AI-based technologies in their clinical practice were assessed. Interviews were performed using either telephone or videoconference based upon pharmacists’ preferences (estimated duration 30 to 40 min). All participants gave their explicit oral or written consent. The interviews were recorded, transcribed verbatim and anonymized.

Because of the COVID-19 pandemic, the focus groups were conducted via videoconference (Zoom Inc.) with a duration of 60 to 75 min. An interview guide was developed and reviewed by co-authors (Appendix 1b). After oral consent, focus groups were recorded, transcribed verbatim and anonymized.

Transcripts were not returned to the participants for correction or comment, and participants did not provide feedback on the findings.

**Quantitative analysis:** A survey was created for the purpose of the study (Appendix 2) collecting patient socio-demographic information (year of birth, gender, education, type of health insurance (n = 4 questions)), clinical information (presence or absence of respiratory symptoms, duration of respiratory symptoms, drug treatments, chronic comorbidities, general health status (n = 19)), satisfaction with healthcare, and perception of digitalization of care (n = 6). The level of satisfaction and the level of trust in AI use in the healthcare domain as well as the level of acceptability of the Pneumoscope™ in CPs were evaluated by scoring (1 (lowest) to 10 (highest score). If interviewed patients were suffering from a respiratory disease, an extended questionnaire was used to collect data on the level of severity of their respiratory disease (n = 5). All documents were co-created in collaboration with a patient partner living with a chronic respiratory disease, who co-designed the questionnaire and consent form, and trained the Master student in the collection of patient data through role play.

### Data analysis

Data analysis of the qualitative study was performed by thematic coding. The analysis of the first four transcriptions of the individual interviews was carried out by two independent investigators (NO and CB) to guarantee a reproducible analysis and identification of emerging themes. The themes were identified through an inductive analysis and were approved by the investigators. The remaining 11 transcriptions were coded by the Master student (NO) based upon previous validation. The same procedure was performed for the focus groups.

The quantitative data was collected into REDCap and descriptive statistics were conducted. Linear regressions were performed on Microsoft Excel 2021 for discrete and continuous variables. The Mann-Whitney test was carried out for binary variables to estimate differences between two groups of variables normally or non-specifically distributed, using Stata 18 software (StataCorp).

### Ethics approval

The Geneva Cantonal Commission on Ethics in Human Research (CCER) confirmed that this quality improvement project did not need to be formally approved by the Ethics Committee (REQ-2021-00766). Then, ethics committee approval was obtained from the ethics committee of the University of Geneva, *Commission Universitaire pour une Recherche Ethique à Genève* (CUREG-2021-08-62).

## Results

### Qualitative sub-study of pharmacists’ acceptability and readiness of the Pneumoscope™

Out of 32 contacted community pharmacists, 15 volunteered for the individual interview (step one) (response rate 88%), two of whom declined subsequently due to time constraints (12%). For the focus groups (step two), five pharmacists from step one (33%) and one pharmacist who had no time for step one participated, resulting in two focus groups of three pharmacists each. In total, 16 pharmacists volunteered for the qualitative sub-study. They were working in 12 different CPs, 62.5% were men with an average of 18 years of experience (Table 1). The pharmacies participating in this study were located in six out of the seven French-speaking cantons of Switzerland. There was a good balance between urban (n = 7) and rural (n = 5) areas and between group (n = 6) and independent pharmacies (n = 5). Data saturation was achieved through 15 individual interviews with pharmacists.

**Table 1.** Characteristics of the interviewed pharmacists and their pharmacies.

		Number (%) (unless specified otherwise)
Pharmacists		16
Sex	Female	6 (37.5)
	Male	10 (62.5)
Years of Experience (median, Q1-Q3)		18 (10–27)
Pharmacies		12
Location of pharmacy	Urban (> 10’000 inhabitants)	7 (58)
	Rural (< 10’000 inhabitants)	5 (42)
Type of pharmacy^a^	Neighbourhood	8 (67)
	Passage	4 (33)
Ownership of pharmacy	Group	6 (50)
	Independent	5 (42)
	Chain	1 (8)

^a^ Type of pharmacy has been self-assessed by the interviewed pharmacists.

Management of all types of symptoms consists mainly in an anamnesis, over the counter (OTC) treatments or orientation of patients in the healthcare system according to the collected information.

### Triage activities in community pharmacies in dyspnea and associated symptoms

The most common symptom of patients presenting to a pharmacy for triage is respiratory discomfort related to dyspnea. Pharmacists reported being rarely confronted with acute severe asthma symptoms. In such a case, the most common patient would be an adult but could also be a child.

“To be honest, it was more likely to be from the thirties on, not before, or parents coming and saying: I have not got salbutamol for my kid, he has had a crisis […]” [P1]

“Well, major attacks [acute asthma] are quite rare, frankly once or twice per year.” [P2]

For chronic asthma, the frequency of visits in CPs is higher, representing an heterogenous patient population. Most pharmacists report that symptom management is not requested in CPs, as this is the responsibility of physicians. All pharmacists agreed that patients solely visit pharmacies for medication dispensing. However, some also mentioned that patients needed further explanation on treatments and/or training in the use of the inhalers, especially at treatment initiation.

“Asthma is a common problem […]. I think that every day we have a prescription for asthma […]” [P8]

“In fact, it's often people on treatment, who come to renew their prescription or to get a new prescription, so I think we're not so much being asked to manage their symptoms, but rather people coming to get their treatment.” [P10]

“To provide them [patients] their medication, they don't expect much from the pharmacist […]” [P4]

For COVID-19, the frequency of visits in CPs varied depending on the period. During the lockdown, crowds overflooded pharmacies (“It was completely out of the ordinary” [P12]), but after the lockdown, the situation calmed down (“Now [in 2021] it's between 1 to 5 cases a day” [P3.2]). Symptoms described by these patients were respiratory symptoms such as cough, breathing difficulty and fever (“breathlessness, difficulty breathing, dry cough and fatigue” [P1]).

All pharmacists agreed that requests for other infectious respiratory diseases were seasonal and that these needs were expressed by all types of patients, regardless of age. The most reported symptoms were coughing or difficulties breathing, and fever.

“So around 30 cases per day in general, 30 to 40 per day in winter and much less in summer. Summer between 1 and 5, often from May to September. From October, November, January we have 50 cases per day, and only over the counter.” [P13]

“All patients, from infants to elderly, but we are not going to look after them in the same way.” [P6]

### Pharmaceutical care in case of dyspnea and associated symptoms, rationale, and potentials

Pharmaceutical care in case of acute asthma, as reported by pharmacists, consists of administrating a short-acting beta2-agonist (SABA)^33^ in a confidential separate office space and trying to reassure the patient. Pharmacists will do an anamnesis including questions related to the nature of the attack and their usual treatment. Pharmaceutical assistance for acute asthma is seen as crucial and an ambulance is considered if the situation cannot be stabilized.

“We invite the patient to the back office, give him/her salbutamol and calm him/her down because the situation often causes anxiety […]. The salbutamol restores normal breathing. Then, we will send him/her to his/her doctor or to the emergency room.” [P1.2]

“The person who is not a known asthmatic, I would say that they really need help, they come to the pharmacy because we are the most accessible place for them and then they are really seeking for us to help him/her to breathe properly again.” [P6]

“They need relief through bronchodilation to be able to breathe normally again. It seems quite logical that they come to us, they are uncomfortable or in real trouble, they want to be relieved quickly!” [P9]

Pharmacists perceive themselves as easy access points of care by complementing medical care when physicians are not accessible or unavailable.

“But as soon as the doctor's office is closed or if the doctor is not available, and we are open, we have to respond to the patient's request, if there is no doctor, we help in emergencies.” [P3]

To support the anamnesis and the management of patients with acute asthma in CPs, pharmacists mentioned several tools such as the oximeter [P5], the inhalation chamber to facilitate SABA administration, and advanced pharmaceutical triage algorithms (the Swiss netCare^®^ pharmaceutical algorithms^34,35^) [P9]. Regarding pharmaceutical care for children with acute asthma, two pharmacists emphasized that this was not their responsibility, while two other pharmacists with several years of experience felt confident in this kind of healthcare services.

In chronic asthma, all pharmacists agreed playing an important role in patient's treatment follow-up and medication adherence, as they accompany long-term treatments and are perceived as being a mediator between patients and prescribers. Pharmacists monitor therapeutic coverage through medication renewals and computerized pharmaceutical patient records. In situations of SABA overconsumption, the pharmacist initiates a discussion with the patient, directs him/her to the doctor or even calls the doctor to discuss the treatment. A pharmacist indicates to increase the dosage of corticosteroid/LABA with the patient in the event of a deterioration, pending medical advice. As for the tools and algorithms used by pharmacists for chronic asthma, a variability in practice was observed. Pharmacists from groups or chains pharmacies mentioned having access to guidelines such as AsthmaCheck^1^, and tools such as the oximeter and peak flow meter but reported not using these frequently.^34^ Fever, respiratory-dependent pain, and the duration of symptoms are elements favouring a medical consultation.

For patients’ management presenting COVID-19 like symptoms, the national recommendations were applied (antigenic test or doctor visit).

### Actual limits of pharmaceutical care in case of dyspnea and associated symptoms

Barriers to acute clinical risk management identified by pharmacists were stress, lack of practical training, lack of supervision of emergencies in the pharmacies (“I don't think there's much supervision of emergencies” [P3.2]) and difficulty in assessing the level of urgency.

“So honestly the stress (…), when they are dying you must take a quick decision and preferably the right one […]” [P1]

“Perhaps we need to get videos presenting the different cases […]. We need something more visual I think, not theoretical, we know that.” [P7]. “A quick analysis and training could be valuable to better understand these emergency situations. [P5]”

The lack of role definition and pharmacists’ responsibility and the lack of guidelines for chronic disease management in CPs is perceived by pharmacists as a limit.

“We don't really know what to do in chronic patient care. We do with our knowledge, our feelings, and our know-how, but we don't have any real instructions or tools to manage them. netCare^®^ [advanced triage in community pharmacies in Switzerland] is a plus, it is true that we could do something much better for chronic asthma.” [P9]

The situation is similar for the management of patients presenting with COVID-19 symptoms, where there are no clear guidelines except for the performance of a nasopharyngeal antigen test.

In general, independent pharmacies felt more deprived in terms of tools for triage than group or chain pharmacies.

### Perspectives in managing common infectious respiratory diseases

Community pharmacists are in high demand for the management of common infectious respiratory diseases. Indeed, a pharmacist expressed the need for a better-defined framework to improve the care of patients with infectious respiratory diseases in pharmacies [P5]. Another pharmacist describes the need to take care adequately of all patients in pharmacies, who do not need to see a physician to limit the overload of medical practices [P12]. A third pharmacist pointed the finger at the lack of training in semiology and diagnosis to rule out red flags [P4]. Note that several pharmacists use telemedicine to resolve red flags.

### Use of AI

Pharmacists agreed that artificial intelligence (AI) devices may support to enhance pharmaceutical services offered in community settings, to strengthen their clinical pharmacy health care provided and to speed up and reinforce confidence in decision making. A pharmacist highlights that AI could enable efficient triage and thus unclog the healthcare system but also allow pharmacists to conduct chronic disease monitoring in the future. However, some pharmacists feel afraid about being replaced by AI in the long-term.

### Pharmacists’ acceptability and readiness to use the Pneumoscope™ in CPs

All the pharmacists included in the focus groups spoke positively about the Pneumoscope™ presented and they perceived that the device could support decision making for the healthcare management and triage of all types of patients with respiratory symptoms. With CPs being one of the first points of entry into the healthcare system for many patients, the Pneumoscope™ would increase the efficiency of triage in pharmacies. A pharmacist admits seeing no need to use the device in all patients but recommends using it specifically in case of triage doubts if the symptoms are suspicious and/or if the patient is vulnerable. The usefulness of the digital device in reducing healthcare costs and workload for GPs was mentioned by two pharmacists.

Facilitators for implementing the Pneumoscope™ were multi-levelled. First, a pharmacist mentioned the shortage of GPs as an open door for the implementation of the Pneumoscope™ in their rural health care context. Second, the ease and speed of use of the Pneumoscope™ was seen as a key benefit. Third, two pharmacists mentioned the possibility of implementing the Pneumoscope™ within their advanced pharmaceutical triage algorithms (for example, netCare^®^^34,35^) and of harnessing the device into a structured interprofessional collaboration, for example during pharmacist-assisted telemedicine, as HUG@Home, an assisted teleconsultation developed in CPs in Geneva.^36^ According to all pharmacists, physician-pharmacist interprofessional consensus is a formal prerequisite for the implementation of the Pneumoscope™ to legitimate the pharmacist's role. Physician-pharmacist quality circles would be a good place to co-construct the use of the Pneumoscope™ in an interprofessional perspective.

Perceived limitations of the use of the Pneumoscope™ in CPs were lack of time if the pharmacist is alone. The most cited barrier was the cost of the service to be paid by the patient and the lack of actual reimbursement for the pharmaceutical service by the healthcare system. Only one pharmacist wondered about the reliability of the device.

## Quantitative substudy–patients’ acceptability

In total, 131 patients were interviewed in the 13 CPs. The majority were women (74%) with an average age of 53 [39,66] years, and 53% had a high level of education (i.e., university or graduate schools). Main reason for visiting their pharmacy was to obtain or renew medications on a prescription (44.8%), 32% respondents suffered from chronic diseases and 15% had chronic or acute respiratory disorders (Table 2).

**Table 2.** Socio-demographics of the interviewed patients and reasons for pharmacy visits.

		Number (%) (unless specified otherwise)
**Total number of patients**		**131**
Female		97 (74)
Age (years) (average; variance; Q1; Q3)		53 ± 16.9 (66; 39)
Level of education	Obligatory schooling	8 (6)
	Apprenticeship	17 (13)
	Higher vocational school	25 (19)
	High school	11 (9)
	Higher education / University	70 (53)
Patients with respiratory troubles		20 (15)
Reasons for pharmacy visit^a^ (N = 145 reasons)	I bring a medical prescription	47 (32.4)
	I pick up self-medication	28 (19.3)
	I come for a COVID-19 test	18 (12.4)
	I come for prescription renewal	18 (12.4)
	I come to ask my pharmacist for advice	15 (10.3)
	I come to pick up COVID-19 auto tests	4 (2.7)
	Other (e.g., parapharmacy, medical devices)	15 (10.3)
Who was the recipient of the visit to the pharmacy?	I come for myself	104 (80)
	I come for a relative	16 (12)
	I come for a child	11 (8)
Patient perception of their health in general	Excellent	25 (19)
	Very good	55 (42)
	Good	35 (27)
	Satisfied	14 (11)
	Bad	2 (1)
Use of smart phones	Use at least once per day	123 (71.8)
	Never	8 (4.6)
**Sub-population of patients with respiratory diseases (n = 20)**		
Number of patients	Without acute symptoms	16 (80)
	With acute symptoms	4 (20)
Types of respiratory disease	Asthma	9 (45)
	Allergic asthma	6 (30)
	Exercise-induced asthma	1 (5)
	Asthma & chronic pneumonia	1 (5)
	Bronchiectasis	1 (5)
	Not provided	2 (10)

^a^ More than one reply per patient was possible.

Most respondents were satisfied with the CP they visited, with 96% rating their satisfaction as 8/10 or higher. Among the 131 interviewed patients, 80% (n = 104) replied that they have come for themselves, 12% (n = 16) for a relative and 8% (n = 11) for a child.

**Figure 2.**
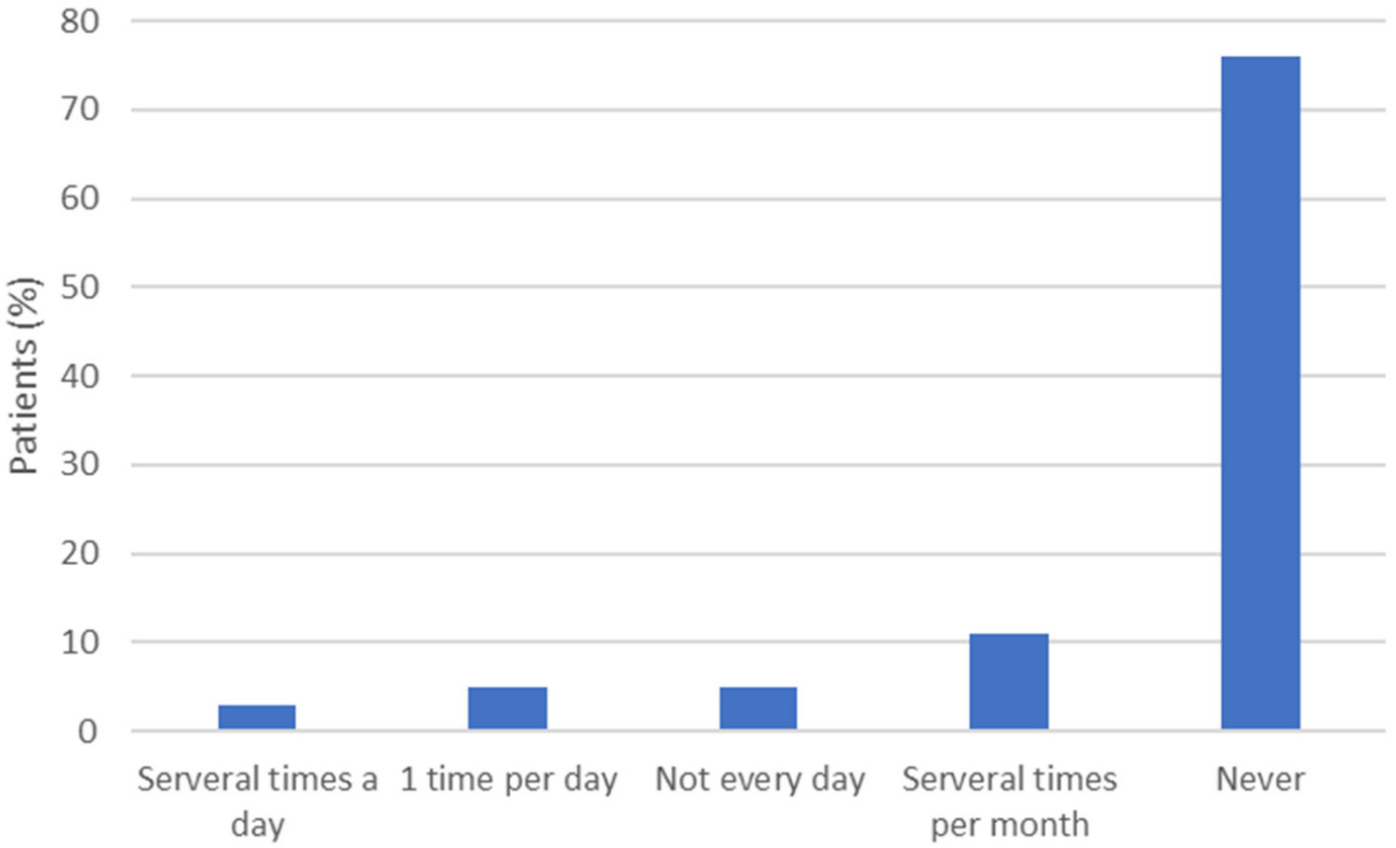
Patients (%) using e-health applications on their smartphone (n = 131).

94% of the patients reported using a smartphone at least once per day while 6% do not use one at all. Figure 2 shows the number of patients using a mobile application in relation to health care, with 76% of the patients not using a e-health application on their smartphone and 3% using them several times per day.

**Figure 3.**
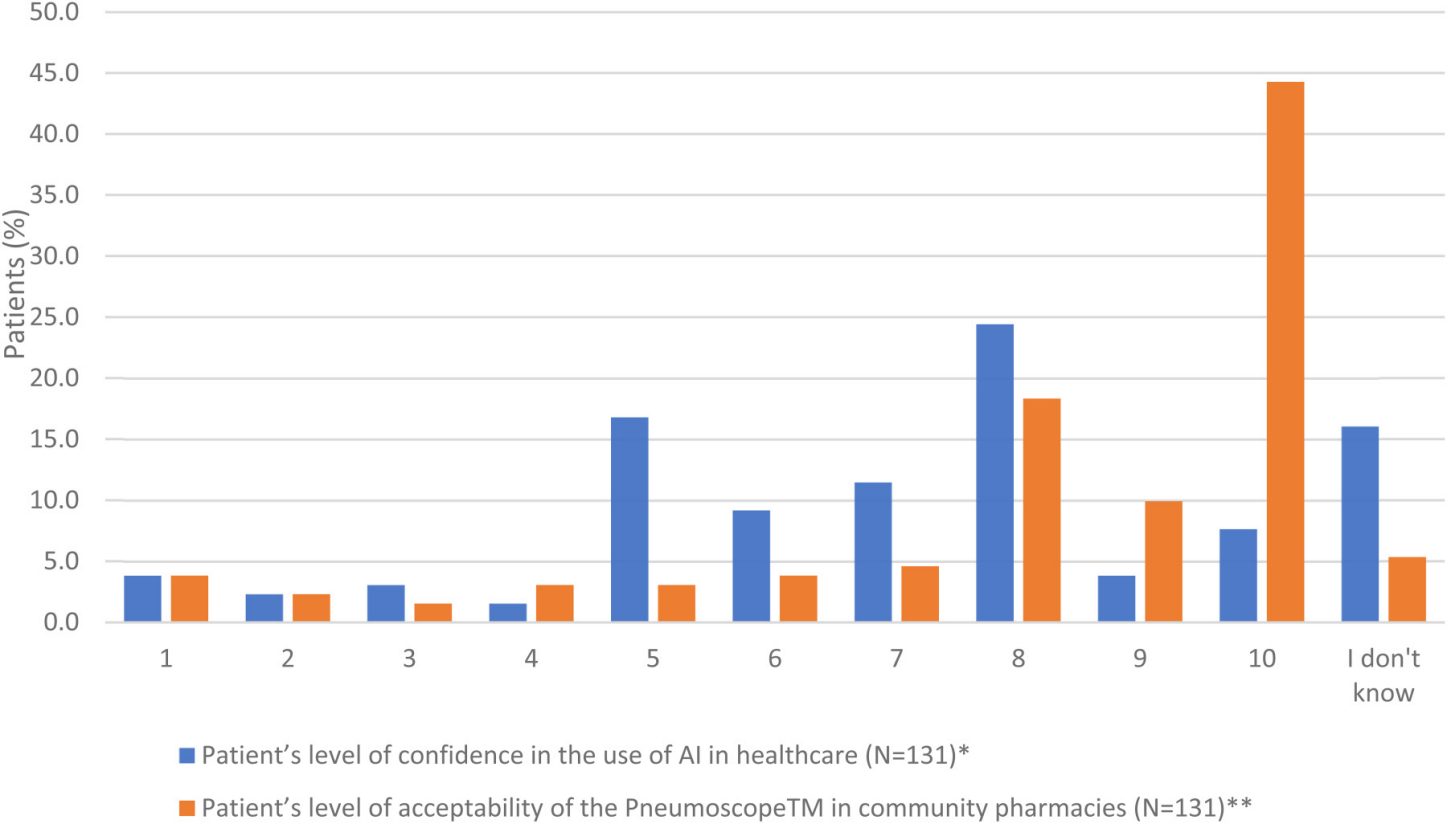
Patients’ level of confidence in the use of AI in healthcare and level of acceptability of the pneumoscope™ use in community pharmacies (score 1 (lowest)-10 (highest))

Patients’ confidence in the use of AI in healthcare and their acceptability of the use of the Pneumoscope™ in CP settings are shown in Figure 3.

*For the patients’ level of confidence in AI: without considering the answers of “I do not know”, the average, median and mode (the most frequently occurring number) are 6, 7 and 8, respectively. The distribution is skewed to the right with an asymmetrical coefficient of −0.67.

**For the patients’ level of acceptability of the Pneumoscope™: without considering “I do not know”, the average, median and mode are 8, 9 and 10, respectively. The distribution is skewed to the right with a coefficient of asymmetry of −1.56.

The reasons of various AI perceptions are listed in Appendix 3.1, where several patients expressed their trust and mistrust. All the comments related to the use of the Pneumoscope™ in CP settings are listed in Appendix 3.2. In case pharmacies would offer such a service, 76% of interviewed patients expressed themselves in favour of reimbursement by the health insurances. The level of patient acceptability to use the Pneumoscope™ is correlated with the patients’ level of confidence in AI (p = 0.0092) and with the location of their CP (p = 0.0276) (Table 3).

**Table 3.** Acceptability of the pneumoscope™ by patients in community pharmacies.

		Patient acceptability of the Pneumoscope™ (1–10, with 10 =highest acceptability)	
Variable		Correlation coefficient (linear regression)	p-value
Year of birth		0.073	0.4193
Level of confidence in artificial intelligence (1–10, with 10 = highest level of confidence)		0.255	0.0092
		N (%)	p-value^a^
Participants … N = 124 (7 = do not know)	With respiratory diseases	17 (14)	0.5471
	Without respiratory diseases	107 (86)	
Participants … N = 101^b^ (5 = do not know)	With chronic diseases	41 (41)	0.8309
	Without chronic diseases	60 (59)	
Participants of pharmacies located… N = 124 (7 = do not know)	In the city centre	47 (38)	0.0276
	In the suburb	77 (62)	

^a^ Mann-Whitney 2-sided test;

^b^ This question was added after the beginning of the study

## Discussion

This study introduces new perspectives for integrating e-healthcare in CPs, focusing on pharmacists’ and patients’ acceptability and pharmacists’ readiness for digital devices like the Pneumoscope™. To our knowledge, this is the first study evaluating patients’ interests in new pharmaceutical services offered based upon an AI-driven stethoscope. The findings are significant in two domains: pharmacists’ perceptions of digital health devices powered by AI for respiratory disease management and patients’ perspectives on these services within CPs. Pharmacists perceived significant potential in adopting AI-driven tools to enhance their role in respiratory disease management. Many pharmacists viewed this digital device as an opportunity to strengthen interprofessional collaboration with physicians and patients, strengthen their legitimacy in patient care, and streamline the triage process. However, the lack of official recognition and reimbursement for such an AI-based pharmaceutical care service remains a critical barrier. Patients’ perceptions showed high satisfaction in general with healthcare provided by CPs. Interviewed patients could imagine using such a digital service in CP settings, however the support of pharmacists will play an essential role in their acceptability of the Pneumoscope™.

Three key themes emerged from the pharmacists’ perspectives. Interprofessionality is considered essential for successful implementation of such a new technology, requiring structured collaboration with physicians and clear role definitions to avoid misunderstandings and competition.^37^ The Pneumoscope™ could serve as a bridge, fostering trust and recognition among healthcare providers. Many pharmacists believe that tools like the Pneumoscope™ will strengthen their role as essential healthcare providers, especially in advanced triage and personalized patient care. However, proper training and interprofessional frameworks are needed to consolidate this legitimacy, as well as well-defined pharmaceutical services. Applications of AI in CP practice are being discussed to extend its impact.^38^ Adequate reimbursement is seen as essential for integrating these pharmaceutical services sustainably. Without it, pharmacists fear that these innovations may be inaccessible to patients or create financial burdens.

Patients expressed high satisfaction with currently delivered CP services, aligning with previous literature that highlights the importance of pharmacist–patient relationships.^39^ They showed openness to using AI-based tools like the Pneumoscope™, especially when supported by healthcare professionals.^40^ The study identified a correlation between patients’ confidence in AI and their acceptability of the device, suggesting that trust in technology plays a pivotal role in adoption.^41^ Contrary to earlier research, this study found no significant relationship between age and acceptance of AI devices.^42^ Instead, geographic location influenced acceptability, with patients in urbanized areas showing higher receptiveness.^16^

The successful implementation of AI tools like the Pneumoscope™ faces several challenges. Medical decisions will be based upon the Pneumoscope™ that falls under the definition of a medical device as per European Union Medical Device Regulation 2017/45 (which also applies to Switzerland) and United States 21 CFR 800-1299. Thus, the medical device needs to be registered and validated as per these regulations and state-of-the-art guidelines. Risks and benefits need to be demonstrated through extensive clinical trials, for example, by demonstrating the comparability between human vs. artificial intelligence results. The device must undergo rigorous validation to ensure safety and reliability, as it will inform critical medical decisions. Pharmacists need robust training to manage emergencies effectively and make confident decisions using AI tools. Building patient confidence requires transparent communication about the device's benefits, limitations, and data privacy measures.^41,43^ Despite these challenges, AI devices such as the Pneumoscope™ present opportunities to enhance CP services.^44^ They can improve the accuracy of triage, reduce the burden on physicians, and empower pharmacists to play a more central role in healthcare delivery.

Due to their accessibility, CPs are an entry point into the healthcare system that patients readily use. AI devices such as the Pneumoscope™ would make it possible to put pharmacists’ skills and knowledge to a greater extent at the service of patients and public health. For successful implementation, several factors must align. Clear role definitions between pharmacists and physicians are essential for ensuring coordinated care.^37^ Clinical and health economic studies must demonstrate cost-effectiveness to enable reimbursements by healthcare insurance. Health insurance coverage for these services is critical to making them accessible and sustainable. Without adequate reimbursement, the cost burden on pharmacies and patients could hinder adoption.

Such digital healthcare services guide patients through the healthcare system according to the severity of the symptoms and support their follow-up, with the potential to improve timely diagnosis. Pharmacists gave favorable opinions about the possible implementation of the Pneumoscope™ in CPs because of its ease of use, the opportunity it offers to answer needs of patients visiting their pharmacy for semi-urgent complaints and to legitimize their professional role in primary care in collaboration with physicians. To increase the impact of the Pneumoscope™, it should be associated to telemedicine assisted by the pharmacist when pathological respiratory sounds require a medical assessment.^36^

This research has some limitations. Firstly, data collection with pharmacists took place during the COVID-19 pandemic, making in-person contact impossible; interviews and focus groups were therefore conducted via digital platforms, using a rigorous interviewing methodology. Secondly, patient enrollment occurred during office hours, meaning no data was collected during evenings, nights, or weekends. This may have influenced why no patient came to the pharmacy for a respiratory urgency. Additionally, certain patient populations working during office hours may be underrepresented, whereas others, such as retired individuals or stay-at-home parents, may be overrepresented. Selection bias may have occurred as only volunteers were recruited, potentially favoring novel pharmaceutical services and reflecting higher confidence than non-participants. However, the diversity of responses collected has been recognized, capturing different perspectives. Few patients with respiratory problems could be interviewed due to the timing of the study during end of summer and early fall when seasonal respiratory diseases were not prevalent. Moreover, the study relied on photo mock-ups of the Pneumoscope™ as no prototype was available, and it was conducted in one Swiss canton with a single interviewer. Repetition of the study will be needed when the device is available and can be assessed in CPs. The study did not explore pharmacists’ technical and operational readiness to integrate the device into their daily workflow, particularly space constraints or technical support needs. Additionally, patients’ preferences regarding whether they would prefer respiratory examinations conducted by a pharmacist or a doctor were not examined. Understanding these preferences could help address any resistance to trust and improve patient readiness. Finally, the study did not investigate patients’ concerns regarding AI and data privacy.

Despite these limitations, AI-driven tools like the Pneumoscope™ have the potential to improve patient navigation within the healthcare system by enabling timely triage and follow-up care. Pharmacists view these innovations as a means to enhance their legitimacy and expand their role in primary care and public health. Coupled with telemedicine, tools like the Pneumoscope™ could revolutionize respiratory care in community settings, offering patients more comprehensive and cost-effective healthcare services.

## Perspectives and conclusion

Community pharmacies are pivotal entry points into the healthcare system due to their accessibility and convenience. AI-devices, like the Pneumoscope™, offer a unique opportunity to put pharmacists’ skills and knowledge to a greater extent at the service of patients with respiratory diseases. However, several factors are essential for the successful implementation of such AI-guided professional activities from scientific validation to demonstrate the device's safety and cost-effectiveness, to the development of interprofessional collaboration for an effective implementation of the Pneumoscope™ and reimbursement by health insurance providers to make these services accessible and sustainable.

AI-driven tools have the potential to improve patient navigation within the healthcare system by enabling timely triage and follow-up care. Pharmacists view these innovations as a means to confirm their legitimacy to be part of interprofessional primary care team. Coupled with telemedicine, AI-devices, such as the Pneumoscope™, could revolutionize respiratory care in community settings, offering patients more timely, comprehensive and efficient healthcare services. Patient adoption is key and needs to be addressed in well-designed implementation studies.

## Supplemental Material

sj-docx-1-dhj-10.1177_20552076241313164 - Supplemental material for Digital healthcare services in community pharmacies in Switzerland: Pharmacist and patient acceptability, and pharmacist readiness–the Pneumoscope™ pilot study Supplemental material, sj-docx-1-dhj-10.1177_20552076241313164 for Digital healthcare services in community pharmacies in Switzerland: Pharmacist and patient acceptability, and pharmacist readiness–the Pneumoscope™ pilot study by Claudine Backes, Coralie Godot, Cédric Yan Gujer, Noémie Obegi, Alexandre Perez, Alain Gervaix and Marie P. Schneider in DIGITAL HEALTH

sj-docx-2-dhj-10.1177_20552076241313164 - Supplemental material for Digital healthcare services in community pharmacies in Switzerland: Pharmacist and patient acceptability, and pharmacist readiness–the Pneumoscope™ pilot study Supplemental material, sj-docx-2-dhj-10.1177_20552076241313164 for Digital healthcare services in community pharmacies in Switzerland: Pharmacist and patient acceptability, and pharmacist readiness–the Pneumoscope™ pilot study by Claudine Backes, Coralie Godot, Cédric Yan Gujer, Noémie Obegi, Alexandre Perez, Alain Gervaix and Marie P. Schneider in DIGITAL HEALTH

sj-docx-3-dhj-10.1177_20552076241313164 - Supplemental material for Digital healthcare services in community pharmacies in Switzerland: Pharmacist and patient acceptability, and pharmacist readiness–the Pneumoscope™ pilot study Supplemental material, sj-docx-3-dhj-10.1177_20552076241313164 for Digital healthcare services in community pharmacies in Switzerland: Pharmacist and patient acceptability, and pharmacist readiness–the Pneumoscope™ pilot study by Claudine Backes, Coralie Godot, Cédric Yan Gujer, Noémie Obegi, Alexandre Perez, Alain Gervaix and Marie P. Schneider in DIGITAL HEALTH

## Notes

1.
https://asthma-check.ch/
