# Supplementary material for: Digital healthcare services in community pharmacies in Switzerland: Pharmacist and patient acceptability, and pharmacist readiness–the Pneumoscope™ pilot study
Source: Digit Health. 2025 Jan 15;11:20552076241313164. doi: 10.1177/20552076241313164 (PMC11736744; doi:10.1177/20552076241313164)
Supplement: sj-docx-1-dhj-10.1177_20552076241313164 - Supplemental material for Digital healthcare services in community pharmacies in Switzerland: Pharmacist and patient acceptability, and pharmacist readiness–the Pneumoscope™ pilot study [file sj-docx-1-dhj-10.1177_20552076241313164.docx]

# APPENDIX 1a:

# Pharmacist interview Guide – Individual (translated in English from French)

## INTRODUCTORY PHASE

**Objective 1** – To better understand the requests made to pharmacies in French-speaking Switzerland by patients presenting with respiratory symptoms that could suggest asthma or cough.

### Acute Asthma: *Individuals presenting to the pharmacy with symptoms of acute asthma*

1. *Do you ever find yourself in this situation? If yes, can you estimate the frequency (per month, or per week)?*
2. *What are the symptoms for which pharmacists are consulted?*
3. *What are the needs of patients coming to your pharmacy for acute asthma?*
4. *Which therapeutic responses do you propose?*
5. *Could you provide some information on the sociodemographic typology of these patients (age, gender, etc.)?*

### Chronic Asthma: *Individuals presenting to the pharmacy with symptoms of chronic asthma.*

1. *Do you sometimes have to treat patients with chronic asthma? If yes, can you estimate the frequency (per month, or per week, etc.)?*
2. *What are the symptoms for which pharmacists are consulted?*
3. *What are the needs of patients who come to your pharmacy with chronic asthma?*
4. *Which therapeutic responses do you propose?*
5. *Could you provide some information on the sociodemographic typology of these patients (age, gender, etc.)?*

### Infectious respiratory disease & COVID

1. *Do you sometimes have to treat patients who present with symptoms that could suggest an infectious respiratory disease (e.g. COVID)?*
2. *If yes, can you estimate the frequency (per month, or per week, etc.)?*
3. *What are the symptoms for which pharmacists are consulted, and which therapeutic responses do you propose?*
4. *What are the sociodemographic typologies of these patients?*

**Objective 2** - To establish the current state of pharmaceutical care for patients with asthma and COVID-19 visiting a community pharmacy.

### Management of acute asthma

*a. Could you describe how you manage patients who come to your pharmacy with acute asthma?*

*b. What tools do you currently use to triage these patients?*

*c. Are they easy to use?*

*d. What are the limits or difficulties you sometimes encounter in this management?*

*e. What would you need to improve care for these patients?*

*f. Do you sometimes have to refer such patients for medical care? If yes, how do you make the decision?*

### Management of chronic asthma

*a. What is the approach to managing patients presenting with chronic asthma?*

*b. Do these patients sometimes not have a prescription or medical follow-up?*

*c. If yes, can you describe how you handle such situations? (Note: facilitate detailed storytelling of a situation that occurred)*

*d. Do you sometimes have to manage symptoms that could indicate a worsening of their asthma?*

*e. What tools are currently available for these clinical situations? Are they easy to use?*

*f. If no tool is available, what would you need to improve the management of chronic asthma?*

*g. Do you sometimes have to refer such patients for medical care? If yes, what do you base your decision on?*

### Infectious respiratory disease & COVID

*a. What is the approach to managing patients presenting with respiratory symptoms (COVID or other)?*

*b. Are tools currently available to triage these patients, and which ones? (If the pharmacist doesn't mention anything, suggest the pulse oximeter or the coronacheck tool)*

*c. Are these tools well established in your pharmacy? Are they easy to use?*

*d. What kind of limits or difficulties do you encounter in these cases (referral to the healthcare system)?*

*e. Do you think the management of these patients in the pharmacy is satisfactory?*

*f. What would you need to improve the management of these patients?*

## PHASE OF RETURN TO REALITY

**Objective 3:** Understand the opinions of Swiss pharmacists regarding the possibility of using new AI-based technologies in the clinical setting in French-speaking community pharmacies.

1. *What do you think about the use of artificial intelligence in your pharmacy?*
2. *What opportunities do you see in the coming years? (Don't be afraid to dream)*
3. *What could artificial intelligence offer?*

## CLOSING OF THE INTERVIEW

*Do you have anything else to add?*

*Expressions of gratitude.*

# APPENDIX 1b:

# Pharmacist interview Guide - Focus Group

# Presentation of clinical scenarios

## INTRODUCTORY PHASE

**Child**

*A mother comes to your pharmacy for the first time with her 5-year-old child, who has a dry cough and difficulty breathing. She asks for cough syrup.*

*How do you handle this situation?*

*What if the patient was known? (unknown acute situation/trust in the relationship)*

**Adult, Acute**

*A 25-year-old patient comes to the pharmacy to get throat lozenges; she has a fever and describes respiratory-related pain.*

*How do you handle this patient?*

*What if she was an unknown patient?*

**Senior, Chronic**

*A 65-year-old patient known to your pharmacy for chronic asthma without any co-treatment, who comes for the second time this month for salbutamol. His medical record also shows a prescription for inhaled corticosteroids.*

*How do you handle this patient?*

*What if he had other treatments (comorbidities)?*

*➔ Presentation of the MOCK-UP Pneumoscope^TM^*

## FOCUS PHASE

**Objective 1** – Understand how the Pneumoscope^TM^ could improve/increase the quality of the management of asthma or other respiratory symptoms in the pharmacy.

1. *According to you, what could the Pneumoscope^TM^ bring to the pharmacy? (Patient orientation, triage, exacerbation management, follow-up management, safety)*
2. *What is the added value of the Pneumoscope^TM^ for patients visiting their pharmacy?*
3. *Overall, what do you think would be the public health added value of the use of the Pneumoscope^TM^ by pharmacists? (Reducing costs, first entry into the healthcare system, reducing the burden on emergency services)*
4. *When would you use this AI-based tool? At what stage of the patient management process?*
5. *What would be the risks for the pharmacist in using the Pneumoscope^TM^? How could these risks be mitigated? (Themes to keep in mind: quality of triage, patient orientation)*

## DEEPENING PHASE

**Objective 2:** to better understand the impact of using the Pneumoscope ^TM^ on the patient journey and on pharmacist-patient/pharmacist-physician relationships.

1. *In your opinion, how would the AI-based Pneumoscope^TM^ be perceived by patients visiting their pharmacy? How would it affect the patient-pharmacist relationship?*
2. *How would the AI-based Pneumoscope^TM^ impact the patient journey through the healthcare system?*
3. *To what extent and how would the use of the Pneumoscope^TM^ in the pharmacy influence the communication and the collaboration between the pharmacist and the medical community?*

## RETURN TO REALITY PHASE

**Objective 3** – Identify initial information to assess the use of the Pneumoscope^TM^ in French-speaking Switzerland by community pharmacists (risk-benefit analysis).

1. *In your opinion, what would be the facilitators and barriers to the use of the AI-based Pneumoscope^TM^ in the pharmacy?*
2. *What new opportunities and benefits would the use of the Pneumoscope^TM^ bring? (Theme to keep in mind: triage and orientation of patients with respiratory symptoms suggestive of asthma or respiratory symptoms)*
3. *What information/guidelines would you like to have to support the use of the Pneumoscope^TM^? (Treatment recommendations, severity of the situation, advice for referral to another care facility?)*
4. *How much do you think your patients would be willing to pay for this type of service? (Price range, reimbursement by health insurance)*
5. *Would you be willing to recommend this tool to other pharmacies?*
6. *What would be your requests regarding the after-sales service of the Pneumoscope^TM^?*
7. *What kind of digital tools or other tests are used by pharmacists, and what are the distribution channels?*

## INTERVIEW CLOSURE

*Do you have anything else to add?*

*Expressions of gratitude.*
