# Supplementary material for: Digital healthcare services in community pharmacies in Switzerland: Pharmacist and patient acceptability, and pharmacist readiness–the Pneumoscope™ pilot study
Source: Digit Health. 2025 Jan 15;11:20552076241313164. doi: 10.1177/20552076241313164 (PMC11736744; doi:10.1177/20552076241313164)
Supplement: sj-docx-2-dhj-10.1177_20552076241313164 - Supplemental material for Digital healthcare services in community pharmacies in Switzerland: Pharmacist and patient acceptability, and pharmacist readiness–the Pneumoscope™ pilot study [file sj-docx-2-dhj-10.1177_20552076241313164.docx]

# APPENDIX 2:

# Questionnaire – Patient perceptions and acceptability of services related to the Pneumoscope^TM^ in the pharmacy

## Clinical Questions

Do you suffer from respiratory problems?

- Yes
- No
- If yes, what respiratory problem do you suffer from?

If "no," do you live with a chronic illness (lasting for 3 months or more, such as diabetes, cardiovascular problems, cancer)?

- Yes
- No
- Comments:
  *If you don't have respiratory problems, answer "others" to question #1 and proceed to question #12.*

1. Why are you visiting the pharmacy today?

- I am consulting my pharmacist for respiratory symptoms or discomfort.
- I have a prescription from my doctor related to current respiratory symptoms.
- I am coming from the hospital and have a prescription related to current respiratory symptoms.
- I am renewing a prescription.
- I am picking up over-the-counter medications.
- Others (explain):
- *If renewing a prescription and if you currently don't have respiratory problems, proceed to question #9.*

1. If you experience symptoms related to respiratory discomfort, can you describe them?
2. Do you have a chronic illness related to your respiratory symptoms/signs?

- Yes
- No
- If yes, which one?

1. On a scale of 1 to 10, how would you rate the intensity of your respiratory symptoms (1 = mild intensity, 10 = strong intensity)?
2. How long have you been experiencing your current respiratory symptoms? *1 day / 2 days / 3 days / 4 days / 5 days / 6 days / 7 days / More than 7 days*
3. Are these symptoms: *New / Frequent / Occasional / Rare*
4. On a scale of 1 to 10, considering your respiratory symptoms, what is your current level of concern?
5. How has your level of concern changed in the last 24 hours? *Not at all / Very little / A little / Moderately / A lot / Extremely*
6. Are you currently taking medications for respiratory symptoms, and if yes, which ones? *(List the medications you remember, and their frequency if applicable (<1, 1, 2, 3, 4, 5, 6 times per week; 1, 2, 3, 4 times per day; Other))*

- Yes
- No
- Comments:

1. In the last 12 months, have respiratory symptoms led you to: (if checked, specify the number of times)

- Medical consultations:
- Emergency room visits:
- Hospitalization:
- Visits to the pharmacy:
- None of these options

1. Do you have other illnesses?

- Yes
- No
- I don't know
- I prefer not to answer
- If yes, which ones?

1. Why did you choose to come to the pharmacy today instead of going to another healthcare facility?

- The pharmacist is close to my home/workplace.
- I prefer to consult the pharmacy/get the advice of my pharmacist first.
- My doctor is not available.
- I don't have a family doctor.
- I don't want to go to the emergency room or a clinic.
- Others
- Comments if others:

1. Would you say that your overall health is: *Excellent / Very good / Good / Satisfactory / Poor*
2. After receiving recommendations by the pharmacist today, do you plan to see your doctor/GP/specialist or to visit the emergency room? *No / Yes / Maybe / Depending on the evolution / I don't know*
3. On a scale of 1 to 10, how satisfied are you with the care you just received at the pharmacy?
4. What made you satisfied?
5. What elements of pharmacy care could be improved?

## Questions about service and digitalization in the pharmacy:

1. Do you use:

- A smartphone (Android or IOS)? Multiple times a day / Once a day / Not every day / A few times a month / Never
- Mobile/digital applications related to your health? *Multiple times a day / Once a day / Not every day / A few times a month / A few times a year / Never*

1. On a scale of 1 to 10, how confident are you in the use of artificial intelligence (limitation of human intelligence through technology) in healthcare? *(1-10 or I don’t know).* Could you explain your answer?
2. On a scale of 1 to 10, if the use of the AI-based Pneumoscope^TM^ was proposed by your pharmacist when visiting your pharmacy for a respiratory problem, to what extent would you accept it? *(1-10 or I don’t know)*
3. For this service, what reimbursement/payment conditions would suit you?

- Only reimbursed by health insurance
- I would agree to pay for it myself.
- No opinion

## Demographic questions:

1. I am coming to the pharmacy today for: *For myself / For a relative / For my child*
2. What is your year of birth?
3. If you come for your child or a relative, what is their year of birth (approximate if not known)?
4. What is your gender?

- Female
- Male
- Other:

1. What is your level of education?

- Mandatory school (primary and secondary)
- Apprenticeship
- Higher vocational school
- Maturity or bachelor's degree
- Graduate schools
- University

1. What is your annual health insurance deductible or that of your relative/child for whom you are visiting the pharmacy today?
2. Pharmacy visits time schedule
3. Do you have any comments?
4. If you/someone you know/your child is currently suffering from respiratory problems, would you agree to be contacted in a week for a 5-minute telephone interview?

- Yes
- No
- I hesitate and will think about it
- I am not currently experiencing respiratory problems
- If "yes" or need 24 hours to think: Name/Phone number/Availability

## Optional phone call one week later:

We met a week ago. You told me about your respiratory symptoms; how have they evolved since then?

- They have completely disappeared.
- They have improved.
- There has been no change.
- They have worsened.

Have you consulted a doctor/emergency room/clinic/hospital/alternative medicine considering the care received? If yes, which professional and when?

Considering the care received, do you have suggestions for improvement/Is there something that could have been done differently?

On a scale of 1 to 10, if the use of the AI-based Pneumoscope^TM^ had been proposed by your pharmacist to accompany the management of your recent respiratory symptoms, to what extent would you have accepted it (according to your current perception)?

## Closing of the interview

Do you have anything else to add?

Expressions of gratitude.
