# Supplementary material for: Digital healthcare services in community pharmacies in Switzerland: Pharmacist and patient acceptability, and pharmacist readiness–the Pneumoscope™ pilot study
Source: Digit Health. 2025 Jan 15;11:20552076241313164. doi: 10.1177/20552076241313164 (PMC11736744; doi:10.1177/20552076241313164)
Supplement: sj-docx-3-dhj-10.1177_20552076241313164 - Supplemental material for Digital healthcare services in community pharmacies in Switzerland: Pharmacist and patient acceptability, and pharmacist readiness–the Pneumoscope™ pilot study [file sj-docx-3-dhj-10.1177_20552076241313164.docx]

# Appendix 3.1 –Patient quotes on perceived trust or mistrust in the use of AI in healthcare (translated from French)

| **Reasons for trust** | **Neutral opinions** | **Reasons for mistrust** |
| --- | --- | --- |
| - General confidence - Belief in modern technologies - Speed and convenience - Facilitates life - Use in the workplace - Promotion of science and innovation - Remarkable/extraordinary - Efficiency/good functioning - Fewer risks of errors - Machines are improving - No emotion, only data - Outperforms human intelligence - Human interpretation is prone to errors - Significant progress - It's reliable - Stability - Provides significant help - Reliability - Precision - AI learns - It's the future - Trust in those developing it | - Lack of knowledge or insufficient knowledge - Never used - Mixed feelings - Many factors to consider (depends on the situation) - Equivalent to humans - Should have the choice - Need for both/complementarity (human and AI) | - Fear regarding the security of computerized data and confidentiality - Ethical concerns - Importance of human contact - Better trust in humans - Mistrust - Replaces jobs - Not a fan of technologies - Too new/not enough experience - Doubts - Makes people less intelligent - Humans can think and feel, not machines - Biased according to developers - Sometimes not well-developed - Fear of robots taking over - Risks of errors - Uncertainty |

# Appendix 3.2 – All patient quotes of the patient interviews regarding the potential use of the Pneumoscope^TM^ in community pharmacies (from positive to sceptical comments) (translated from French)

| **Positive quotes** |
| --- |
| - If it saves me from going to the doctor, that's good. - I think it might even be an advantage if we could have the device at home. - The device is a good idea. - If it's good, then I completely accept. - I find pharmaceutical services very convenient, especially since it's readily available. - Testing out of curiosity. - We have nothing to lose by trying the device; it's good. - I would give the opportunity to try the device. |
| **Neutral quotes** |
| - In my case, there's nothing to be done; there's no treatment, and I've already been diagnosed. The device wouldn't be useful for me, but I think it's a good idea for people without chronic diseases. - I would be okay using the device in case of an emergency. - I prefer the regular stethoscope. - I would accept the device if it was recommended and there was an interest in it compared to going to the doctor. I remain sceptical. |
| **Negative quotes** |
| - I don't think I would have the reflex to go to the pharmacy. As soon as I have a respiratory problem, I quickly go to the doctor because my dad died of lung cancer, so my first reflex is to call the doctor. - If I have respiratory discomfort, I go straight to the doctor. - At first glance, it's a good idea but dangerous because the pharmacist is not a doctor and, therefore, doesn't know the lungs well. - I have difficulty trusting the pharmacist, and I choose my doctor based on various factors (naturopath). - It bothers me that it's a pharmacist who uses the device; for me, it should be the doctor. |
